# Relating Workaholism to Job Stress: Serial Mediating Role of Job Satisfaction and Psychological Capital of Nurses in Angola

**DOI:** 10.3390/nursrep15020043

**Published:** 2025-01-27

**Authors:** Rosa Lutete Geremias

**Affiliations:** 1 Lisbon Accounting and Business School, Instituto Politécnico de Lisboa, 1069-035 Lisbon, Portugal; rhgeremias@iscal.ipl.pt; 2 Centro de Administração e Políticas Públicas, 1300-663 Lisbon, Portugal

**Keywords:** workaholism, job satisfaction, psychological capital, job stress, nursing, Angola

## Abstract

**Background/Objectives**: Previous studies conducted in sub-Saharan African countries have concentrated on examining the challenges of nursing training and the organizational commitment of healthcare professionals, with little attention paid to exploring the mechanisms that contribute to reducing nurses’ job stress. Consequently, the present study addresses a significant gap in the literature by offering an overview of the factors contributing to understanding job stress among nurses in Angola. This study aimed to analyze the direct and indirect relationship between workaholism and job stress with job satisfaction and psychological capital mediating this relationship. **Methods**: Using the quantitative methodology with a cross-sectional design, a questionnaire was administered to 340 nurses (172 men and 168 women). **Results**: The results confirmed that workaholism is positively related to job stress and that job satisfaction and psychological capital serially mediate the relationship between workaholism and job stress. **Conclusions:** These findings highlight the importance of fostering job satisfaction and psychological capital by establishing favorable work environments and promoting nurses’ physical and emotional well-being. In addition, these results may encourage healthcare leaders to create well-designed break areas for nurses to take restorative breaks.

## 1. Introduction

Nursing is widely described as a stressful job, considering that it is associated with excessive working hours due to the demands of caring for patients who require sensitive healthcare [1]. The study performed in Ghana using an occupational stress scale concluded that the stress level in nurses was 29% above the normal level compared to the general population [2]. In developing countries, pressure on nurses to provide sensitive healthcare has increased levels of job stress [3]. According to Hassard et al. [4], job stress was related, on average, to 80% of productivity losses. Thus, previous research has considered identifying mechanisms to reduce nurses’ job stress levels essential [5].

According to Amu et al. [6], African countries have faced challenges in the working conditions of nurses due to the lack of resources allocated to the healthcare sector and difficulties in recruiting qualified and experienced healthcare professionals. In general, nurses in Africa have demonstrated high stress levels, which has harmed their performance and the quality of patient care [7]. The study performed in Uganda with 498 participants from six health units concluded that 71% of nurses presented stress levels considered chronic [8]. Angola is one of the African countries where nurses are confronted with considerable workloads due to the healthcare sector’s severe shortage of human resources [9].

Nurses are the frontline professionals and, as such, are often exposed to stressful situations related to patients, which has led to symptoms of post-traumatic stress disorder and emotional exhaustion [10]. Although the factors that lead to job stress have been largely ignored in studies related to nurses in Africa, it is essential to note that theoretical research does not consider stress to be a spontaneous phenomenon [11]. Consequently, workaholism was identified as a predictor of job stress [12]. For Clark et al. [13], workaholism is a driver of various adverse outcomes, such as the worsening of employees’ physical and mental health. Given the shortage of healthcare professionals in sub-Saharan African countries, the workforce must increase by 140% to meet international health development goals [14]. Contracted professionals have reported the need to work incessantly to provide patients with the necessary life-saving interventions, dramatically increasing job stress [15].

The studies conducted in Angola have focused on analyzing the challenges for nursing education and the organizational commitment of healthcare professionals [9]. According to Sen et al. [16], job satisfaction and psychological capital can contribute to minimizing job stress. However, there is no clear evidence of a study performed with nurses in an African context that has directly analyzed the role of job satisfaction and psychological capital on job stress. Nevertheless, some researchers have highlighted the importance of examining the relationship between workaholism and job stress, e.g., [12]. In studies related to healthcare professionals, we have only seen a few attempts to analyze the mediating role of psychological capital in the relationship between burnout and job stress in Korean psychiatric nurses [17].

In light of the above, the present study contributes to filling the gap in the literature by analyzing the mediating role of job satisfaction and psychological capital in the relationship between workaholism and job stress among nurses in Angola. The present study aims to (1) analyze the direct and indirect relationship between workaholism and job stress and (2) assess the mediating role of job satisfaction and psychological capital in the relationship between workaholism and job stress. The rationale for this study is based on findings from previous research that identified a correlation between workaholism and job stress [13] and studies that demonstrated that psychological capital and job satisfaction can mitigate job stress [16].

According to García-Tudela et al. [18], a healthcare system with acceptable quality standards contributes to its professionals’ low stress levels. It is well established that highstress levels can lead to negative health consequences, such as physical and psychological disorders, which in turn affect patient care [10,19]. Therefore, understanding the contributing factors to minimize nurses’ stress is crucial, as it makes it possible to assess discrepancies in improving the quality of patient care.

This article is organized as follows. First, the definitions of the constructs under study, namely workaholism, job satisfaction, psychological capital, and job stress, are presented, followed by the rationale of the hypotheses. Second, the methodological options are described, such as study design, participants, instruments, and data analysis. Third, the study results are analyzed, followed by a detailed discussion of the theoretical and practical implications. Finally, a brief conclusion of the study is presented.

### 1.1. Theoretical Framework

#### 1.1.1. Workaholism

Scott et al. [20] defined workaholism as an excessive need to be involved in work beyond organizational requirements, which results in a lack of engagement in other significant activities in one’s daily life. Workaholism has been linked to different outcomes, such as mental health problems [21], Job Burnout [22], Career calling [23], turnover intention [24], and work–family conflict [25]. In terms of mental health, a cross-sectional study of 16,426 workers showed positive and statistically significant correlations between workaholism and symptoms of psychiatric disorders such as anxiety and depression, attention-deficit/hyperactivity disorder and obsessive-compulsive disorder [21].

#### 1.1.2. Job Satisfaction

Job satisfaction can be defined as an emotional state, a set of thoughts, and beliefs that individuals develop about their work, and produces positive effects on organizational outcomes [26]. In the healthcare field, previous studies have demonstrated a negative relationship between job satisfaction and burnout among nurses in urology departments in Poland [27], and intention to leave among psychiatric nurses in Jordan [28]. In Africa, specifically in Namibia, South Africa, Zimbabwe and Cameroon, evidence was found of a positive relationship between nurses’ job satisfaction and different outcomes in organizational settings, such as employee engagement [29], authentic leadership [30], and employment equity [31].

#### 1.1.3. Psychological Capital

Psychological capital is composed of the following four psychological capabilities: (1) self-efficacy: which consists of individual confidence that contributes to performing challenging tasks; (2) hope: related to the perseverance necessary to define goals and design the necessary steps to achieve success; (3) optimism: consists of developing positive expectations in the accomplish of future events; and (4) resilience: consists of the individual capability that allows one to recover in the face of adversity to achieve the desired success [32]. For Youssef-Morgan and Luthans [33], the synergistic action of these psychological capabilities allows the achievement of different positive organizational outcomes.

#### 1.1.4. Job Stress

Studies in the job stress area indicate that when the employee identifies a factor perceived as unpleasant or alarming, it triggers a negative response. Therefore, when these factors exceed the employee’s ability to adapt, divergent responses to stress occur [34]. According to Kim and Jung [35], job stress has been identified as one of the main concerns of hospital managers, considering that employees with high stress levels tend not to identify with the values and culture of providing the best healthcare to patients. However, in Africa, studies on job stress have mainly been conducted outside the hospital context and have focused on analyzing the relationship between job stress and other variables such as affective commitment, organizational support, and turnover intention [27].

#### 1.1.5. Hypothesis Development

##### Workaholism and Job Stress

For Lin et al. [36], the stress caused by long working hours among healthcare professionals has encouraged the formation of public policies to limit working hours. For example, Angola implemented an overtime limit of 42 h per month for nurses, diagnostic technicians, and hospital support staff [37]. However, with the advent of COVID-19, reducing working hours has become a challenge, resulting in a drastic increase in work overload, with nurses having to work incessantly for long hours, making it difficult to provide quality healthcare [38]. The high number of working hours among healthcare professionals contributes to increased levels of job stress [39]. Based on the theoretical vision presented by Salama et al. [40], we substantiate the relationship between workaholism and job stress. Given this, the following hypothesis is presented:

**H1:**  *Workaholism is positively related to Job stress*.

##### The Mediating Role of Job Satisfaction Between Workaholism and Job Stress

Job satisfaction is related to an individual’s attitude toward an emotional experience at work. Therefore, all work issues are likely to affect job satisfaction [41]. According to Andreassen et al. [42], workaholism results from compulsive and excessive work, which can negatively impact job satisfaction. A literature review showed that workaholism negatively correlates with job satisfaction [43]. Job stress results from an adverse reaction to unsatisfactory factors that exceed the resolution capacity of the parties involved in the healthcare process [34]. According to Autin et al. [44], when employees are satisfied with issues related to their work, job stress tends to decrease naturally. Therefore, this study suggests that workaholism is negatively related to job satisfaction, and job satisfaction negatively affects job stress. Given this kind of previous conclusions, the following hypothesis is described:

**H2:**  *Job satisfaction mediates the relationship between workaholism and job stress.*

##### The Mediating Role of Psychological Capital Between Workaholism and Job Stress

Psychological capital is a driving force that reduces various adverse outcomes in the organizational field [45]. Some studies suggest that workaholism creates a high-stress environment, which often leads to a decrease in psychological capabilities [46]. For example, the research conducted with 400 teachers and staff in the United States showed a negative relationship between psychological capital and workaholism [47]. Job stress encourages negative reactions to unpleasant or alarming work situations [34]. Psychological capital is the set of capabilities that reflect the employee’s psychological state of development [48]. Therefore, nurses who develop specific psychological capabilities are more likely to adapt to challenging situations and thus reduce their job stress levels. Given this, the following hypothesis is presented:

**H3:**  *Psychological capital mediates the relationship between workaholism and job stress.*

##### Serial Mediating Role of Job Satisfaction and Psychological Capital Between Workaholism and Job Stress

**Figure 1:**
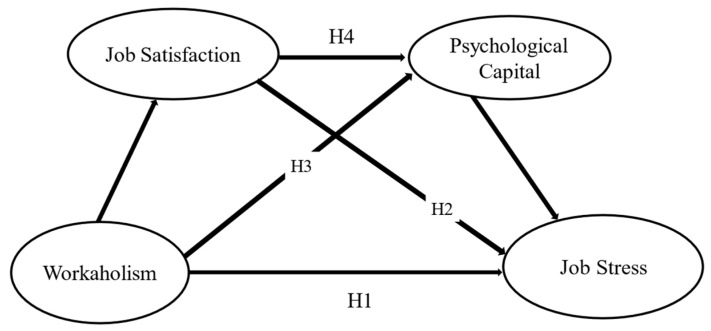
Hypothesized model.

Previous research has shown that when nurses are satisfied with their work, they perform defined tasks with a positive psychological state [49]. This positive attitude towards work reduces undesirable behaviors, such as job stress [50]. Furthermore, when nurses are satisfied with their work, they produce a pleasant emotional response to the challenges presented in performing defined tasks, thus allowing them to overcome uncertainties related to success in achieving defined objectives and future goals [51]. Larson and Luthans [52] demonstrated a statistically significant relationship between psychological capital and job satisfaction. Therefore, the present study assumed that workaholism reduces job satisfaction, increasing psychological capital, which is negatively related to job stress. Figure 1 illustrates the hypothetical model. Based on this evidence, the following hypothesis was formulated:

**H4:**  *Job Satisfaction and Psychological capital are sequential mediators in the relationship between workaholism and job stress.*

## 2. Materials and Methods

### 2.1. Study Design

Quantitative methodology was used to analyze the relationship between the main variables under study. The perspective used was descriptive with a correlational design that included a cross-sectional survey of information among participants. The nurses were selected by convenience using the non-probabilistic method. This option allowed the selection of nurses providing healthcare services in Angola. The non-probabilistic convenience approach offers more straightforward access to nursing staff in a hospital setting, where time constraints are a significant issue due to the high volume of patients [53].

### 2.2. Procedure and Sample

In May 2024, an online self-reporting survey was available on Google Forms. The questionnaire was distributed through personal contacts, on social media and by the managers of different hospitals in Angola. All the nurses were informed of the confidential nature of the information provided, which the application of an anonymous questionnaire would guarantee, so it would not be possible to identify the participants individually. It is also noteworthy that the questionnaire provided a succinct overview of the study’s objectives and the nature of the research. Furthermore, the introductory segment of the questionnaire presented a declaration indicating that, by completing the questionnaire, the nurse was granting informed consent to participate in the study.

Specific settings were implemented in Google Forms to prevent participants from accessing the questionnaire on more than one occasion. Consequently, participants were required to log in to Google, thus allowing a single Google account to respond to the questionnaire on only one occasion. For the data collection process, after obtaining 340 responses, the questionnaire was downloaded in Excel format, which was then coded and imported into SPSS for data processing.

The expectation was to reach 600 nurses from different healthcare services in Angola. However, 340 questionnaires were received, indicating an estimated acceptance rate of 57%. Specifically, responses were received from 28 public and private healthcare institutions (ranging from 8 to 13 nurses per institution). The number of study participants (340) is below the cut-off value of 200 participants for structural equation modelling analysis with maximum likelihood estimation, recommended by Hair et al. [54]. Additionally, we used the G*Power software (version 3.1) to determine the minimum sample size appropriate for the present study. For the medium-size effect, we considered 124 participants (f^2^ = 0.15), an alpha of 0.05, with 95% power, using linear multiple regression, fixed model, and R2 increase. The results show that at least 253 participants would be necessary for testing the research hypotheses. In the total sample, 50.6% were males, and the average age was 35 years (SD = 5.4). Concerning the most significant educational qualifications, 70.2% had obtained a university degree, while 18.8% had completed a master’s degree. On the other hand, it is important to highlight that 79.4% of the nurses had been working for more than four years.

### 2.3. Measures

Workaholism: The 7-item scale developed by Andreassen et al. [42] was used. The sample items include: “I have thought about how to get more time to work” and “I have worked to the point where it’s damaging my health”. The response scale used is a five-point Likert scale ranging from (1) “Totally Disagree” to (7) “Totally Agree”. Cronbach’s alpha presented in the literature was 0.81 [42], and the Cronbach’s alpha calculated for this study was 0.74.

Job Satisfaction: The questionnaire with 5 items, presented by Judge et al. [55], was applied. It is a 5-point Likert scale, from (1) “very dissatisfied” to (5) “very satisfied”. The sample items include “I am enthusiastic about my work” and “I find real pleasure in my job”. The Cronbach’s alpha identified in the literature was 0.92 [55], and the alpha calculated in this study was 0.81.

Psychological capital: The adapted version of the 24-item questionnaire presented by Luthans et al. [32] was used. The scale is comprised of four sub-scales with six items each, corresponding to positive psychological capabilities that assess, respectively, optimism (e.g., “When things are uncertain for me at work, I usually hope for the best”); hope (e.g., “I currently try to achieve my goals with great energy”); self-efficacy (e.g., “I feel capable of helping to set goals/objectives for my hospital”); resilience (e.g., “I always try to manage difficulties at work well, in one way or another”). Responses used a six-point Likert scale, ranging from (1) “Strongly disagree” to (6) “Strongly agree”. The 24-item questionnaire in the original study by Luthans et al. [32] has a Cronbach’s α of 0.89. The Cronbach’s alpha value of the psychological capital scale calculated in this study was 0.76. Furthermore, it is noteworthy that the Cronbach’s alpha values of the psychological capital subscales, as determined in this study, are as follows: self-efficacy (0.74), hope (0.77), resilience (0.72), and optimism (0.78).

Job stress: The 13-item scale developed by Parker and Decotiis [56] was used. The response scale used a five-point Likert type, from (1) “Strongly disagree” to (5) “Strongly agree”. An example item was “My job asks too much of me than it should”. The Cronbach’s alpha value reported by the authors in the literature was 0.86 [56], and Cronbach’s alpha in this study was 0.88.

The scales were translated from English to Portuguese using the translation/retroversion method. The original items and their translated versions were meticulously compared. Two experts, one a native English-speaking teacher and the other a Portuguese English linguistics teacher, were consulted to assess the accuracy of the translation.

### 2.4. Data Analysis

Data analysis was performed in several stages. First, SPSS software (v.29) was employed to perform mean and standard deviation and analyze the internal consistency of the scales. Second, the Amos software (v.29) allowed us to evaluate the factorial composition of all scales. To evaluate the model fit, we used the parameters widely known in the literature presented by Hair et al. [54]. The parameters Tucker–Lewis Index (TLI), Comparative Fit Index (CFI) and Goodness of Fit Index (GFI) must present values equal to or greater than 0.9. While the parameters Standardized Root Mean Square Residual (SRMR) and Root Mean Square Error of Approximation (RMSEA) must have values equal to or less than 0.08. On the other hand, the test of all hypotheses was performed using the Amos software (v.29).

### 2.5. Assessing Common Method Bias

A cross-sectional study is a research design that collects data from a single source over a specific period [57]. This approach is a potential source of common method bias, which can be mitigated through statistical “remedies” [58]. Previous studies have used statistical “remedies” to address common method bias, e.g., [59]. To ensure the data were free from the negative effects of common method bias, this research employed a single-factor test, which yielded a score of 27 for the first factor. This value should be less than 50% [60]. Therefore, common method bias is not a concern for the validation of the results.

## 3. Results

### 3.1. Descriptive Statistics

As proposed by Flake and Fried [61], the answer engagement was analyzed by calculating the standard deviation of answers and the number of items with missing data responses. Consequently, four surveys were excluded because they exhibited more than four missing values and a standard deviation of less than 0.5, as recommended by Esteves et al. [62]. Following the screening process, 336 questionnaires were validated. The data normality analysis was conducted by calculating skewness and kurtosis. The skewness and kurtosis values were within the range of ±2, indicating that all constructs met the normal distribution requirement recommended by Tabachnick and Fidell [63]. Conversely, the multicollinearity test was evaluated by calculating the variance inflation factor (VIF). The results demonstrated that the VIF values were below the cut-off value of 3.3, ranging from 1.050 to 1.086. This indicates the absence of multicollinearity in the data, as previously highlighted by Hair et al. [54].

Table 1 presents the results of the mean, standard deviation, correlation between the variables under study, and Cronbach’s alpha values. The mean values ranged from 2.47 to 3.92, while the standard deviation ranged from 0.67 to 1.06. On the other hand, the results showed that workaholism is positively correlated with job stress (r = 0.310, *p* < 0.01) and negatively correlated with job satisfaction (r = −0.229, *p* < 0.01) and psychological capital (r = −0.142, *p* < 0.01). Job satisfaction is positively correlated with psychological capital (r = 0.195, *p* < 0.01) and negatively correlated with job stress (r = −0.774, *p* < 0.01). Lastly, psychological capital is negatively correlated with job stress (r = −0.211, *p* < 0.01). It is worth noting that all variables under study have Cronbach’s alpha values above 0.70. It is also important to note that, for the purposes of this study, psychological capital was employed as a second-order construct. This is because using a second-order construct allows for more robust results than would be obtained by analyzing the subdimensions separately [64].

Furthermore, a confirmatory factor analysis was conducted to assess the construct validity of all study variables. Table 2 presents the results of the confirmatory factor analysis.

### 3.2. Hypothesis Tests

Hu and Bentler [65] indicated that the hypothesized structural model should have acceptable fit indices. Therefore, our structural model meets the recommended standards. (*χ*^2^ (103) = 208.200, *p* < 0.001; TLI = 0.953; CFI = 0.916; GFI = 0.931; SRMR = 0.080; RMSEA = 0.069). The bootstrap approach was performed to test the hypotheses, with a 90% confidence interval over the standardized indirect effects, which has been widely used in previous studies, e.g., [62].

The results showed a positive and statistically significant relationship between workaholism and job stress (β = 0.136; ρ < 0.01), supporting H1. The second hypothesis is related to the mediating role of job satisfaction in the relationship between workaholism and job stress. The results indicated that workaholism is negatively related to job satisfaction (B = −0.229; *p* < 0.001), and that job satisfaction is negatively related to job stress (B = −0.633; *p* < 0.001). Additionally, the results showed that job satisfaction mediates the relationship between workaholism and job stress (indirect effect = 0.170; 90% CI; LLCI = 0.096; ULCI = 0.233), supporting hypothesis H2.

The third hypothesis suggested that psychological capital mediates the relationship between workaholism and job stress. The results showed that Workaholism is negatively related to psychological capital (B = −0.103; *p* < 0.001) and that psychological capital is negatively related to job stress (B = −0.048; *p* < 0.01). Furthermore, the results confirmed that psychological capital mediates the relationship between workaholism and job stress (indirect effect = 0.024; 90% CI; LLCI = 0.007; ULCI = 0.048), thus confirming hypothesis H3.

**Figure 2:**
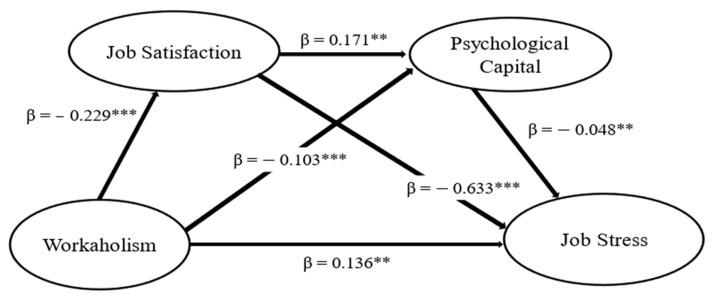
Final model. ** *p* < 0.01 and *** *p* < 0.001.

Finally, hypothesis H4 suggests that job satisfaction and psychological capital serially mediate the relationship between workaholism and job stress. The indirect effect of workaholism and job stress through the mediation of job satisfaction and psychological capital is statistically significant (B = 0.197; LLCI = 0.103; ULCI = 0.237). Therefore, the results showed that there is a negative relationship between workaholism and job satisfaction (B = −0.229; *p* < 0.001), job satisfaction is positively related to psychological capital (B = 0.171; *p* < 0.001), and psychological capital is negatively related to job stress (B = −0.048; *p* < 0.01). These findings support hypothesis H4. The results presented allowed us to validate all the hypotheses defined. Figure 2 shows the results of the hypothesis test.

## 4. Discussion

This study aimed to analyze the direct and indirect relationship between workaholism and job stress, with job satisfaction and psychological capital mediating this relationship. The study supported the positive relationship between workaholism and job stress (H1: workaholism is positively related to Job stress). This finding aligns with previous studies, which indicate that excessive concern about work leads to increased effort and time in performing tasks to the detriment of other important areas of life, contributing to increased job stress [21]. The positive relationship between workaholism and job stress seems to happen because workaholism has been identified as a negative psychological state and chronic pattern in the organizational context that requires a high investment in work, as an indiscriminate and excessive concern for work over some time [66].

For Babapour et al. [1], nursing is a highly stressful profession due to the need to work hard to respond to complex tasks without these professionals having greater authority and a compatible level of responsibility. The study conducted with 300 nurses in Iran showed that excessive workload was associated with problems such as sleep disturbance and job stress [67]. According to Mensah [68], job stress negatively impacts employees’ physical and mental well-being, affecting their intellectual, emotional, and physical balance and ability to achieve the desired performance when performing defined tasks. As the level of job stress increases, its negative effects become more pronounced, potentially reducing job satisfaction, organizational commitment, engagement and organizational citizenship behaviors, inevitably leading to the loss of the organization’s competitiveness [69].

The mediating role of job satisfaction in the relationship between workaholism and job stress was confirmed (H2). This relationship seems to happen because job satisfaction means that tasks are performed with motivation and happiness, resulting in less stress at work [44]. Haji Matarsat et al. [70] pointed out a negative relationship between job satisfaction and work–family conflict among nurses from the largest referral hospital in Brunei. Workaholism is considered a relevant source of stress among nurses, so when workaholism increases, the pressure to perform defined tasks promptly is more significant [66]. A study of healthcare professionals, after 79 observations for a total of 316 h with 15 registered nurses, showed that nurses were required to provide more patient-centered healthcare and that work was not evenly distributed among nurses, with a 12 h shift as a reference, which contributed to an increase in job dissatisfaction and stress [71]. Furthermore, the study conducted by Zheng et al. [72] showed that job satisfaction was lower for those who worked more than 9 h, and the impact on reducing job satisfaction was even more significant after 12 h of work.

The third hypothesis (H3), which proposed the relationship between workaholism and job stress through psychological capital was confirmed. For Yen et al. [71], with technological innovations and the growing search for competitive advantages, employees have been increasingly encouraged to work longer hours and deliver results beyond expectations, making it more difficult to separate personal and professional life. The increase in nurses’ working hours brought about by COVID-19 has meant that these professionals have acquired an uncontrollable need to work to provide the best service to the patient and contribute to saving lives, thus creating undesirable results, such as job stress [7]. The study by Moyer et al. [47] showed that workaholics are likely to have weaknesses in their physical and emotional well-being, which could negatively affect their psychological capabilities. Psychological capital represents a set of psychological capabilities that allow the employee to cope with adverse situations and achieve the expected success, which reduces job stress [45].

Previous empirical studies performed in the healthcare sector have highlighted the relevant role of psychological capital in achieving better results. A cross-sectional study of 575 nurses from two central hospitals in Cameroon found that psychological capital was positively related to job performance [73]. Additionally, a study performed with 339 nurses in China from different areas (gastroenterology, endocrinology, respiratory medicine, cardiology, nephrology, neurology, radiation therapy and geriatrics), concluded that psychological capital allows for improving the capacity for humanistic care [74]. On the other hand, the negative relationship between psychological capital and burnout has been reported in the literature [75], as well as turnover intention [76].

The serial mediating role of job satisfaction and psychological capital in the relationship between workaholism and job stress was supported (H4). This seems to happen because nurses’ workload increases substantially in sensitive work areas with a high-risk level of contagion, and their job satisfaction decreases [39]. For Amu et al. [6], the decline in job satisfaction in African countries becomes critical when nurses are overly concerned with providing patients with quality health services. However, the hospitals do not provide improved working conditions and cannot attract enough health professionals to meet the high demand for healthcare. Regarding the relationship between job satisfaction and psychological capital, previous research has empirically shown that job satisfaction is positively related to psychological capital, e.g., [52]. In addition, psychological capital decreases negative outcomes such as job stress [45]. Therefore, both job satisfaction and psychological capital can buffer the impact of workaholism on job satisfaction.

Contrary to what is widely known in the literature that nursing is a predominantly female profession, the Angolan context seems to challenge this assumption. According to Teixeira [77], there are several obstacles to women’s access to and retention in higher education. (1) Women are taught and coerced to assume responsibility for domestic tasks and the care of their families, which contributes to an increase in school dropout rates; (2) the high number of early pregnancies leads to high levels of discrimination and prejudice in the school context, thus contributing to the reduction in the academic success rate among women. Therefore, the low level of literacy and formal technical education in different areas, and nursing is no exception, means that men mostly perform formal employment. Therefore, to generalize these results, it is necessary to keep this reality in mind.

### 4.1. Limitations and Future Directions

The present study has some limitations. Due to limitations in accessing more detailed information about nurses’ work schedules, this study did not consider the definition of a cut-off score for categorizing workaholics, as presented by Andreassen et al. [42]. Future studies should explore this alternative path. Second, using the same data collection source increases the common method bias [59]. Future studies should use different data collection sources, as suggested by Hulland et al. [58]. Third, we used a non-probability convenience sampling method, which may compromise the generalization of the study’s results, as pointed out by Li et al. [78]. Therefore, future studies may apply stratified probabilistic sampling, as suggested by Martínez-Mesa et al. [79]. Furthermore, considering the objectives defined in this study, only psychological variables were included in the final model, excluding sociodemographic variables such as age and sex. This option was adopted given that evidence on the role of sociodemographic variables in healthcare is scarce and contradictory [80]. However, future studies are encouraged to address this issue. Finally, the nursing sector in Angola is not properly stratified (e.g., primary, secondary or tertiary healthcare institutions) due to the disruption registered in the national healthcare system and the reduction over the years in health coverage caused by the armed conflict that the country has experienced [81]. This fact is a constraint for the generalization of the results of this study.

### 4.2. Study Implications

This study has the following theory and practice implications. First, we present evidence that workaholism is related to job stress. Given this, public managers should develop realistic policies that limit working hours without compromising the normal functioning of hospitals and the quality of services to patients. In addition, these findings may encourage healthcare leaders to create well-designed break areas for nurses to take restorative breaks [82].

Second, there is evidence of the mediation role of job satisfaction in the relationship between workaholism and job stress. This information reinforces the primary role of job satisfaction in achieving various positive individual and organizational outcomes [44]. This study enhances the importance of promoting job satisfaction by creating good working conditions and an environment that increases nurses’ physical and emotional well-being. Furthermore, this study provides an overview of how hospital managers can contribute to reducing nurses’ job stress. On the other hand, nurses and other healthcare professionals should see this work as a core objective of improving their mental and physical health.

Another contribution relates to the notion that job satisfaction and psychological capital mediate the relationship between workaholism and job stress. These findings fill an essential gap in the literature, which has led some researchers, such as Betke et al. [5], to highlight the importance of finding solutions to address nurses’ job stress in different contexts. For Amu et al. [6], it is crucial to understand nurses’ job stress, especially in countries with deteriorating working conditions and scarce resources allocated to the healthcare system. Finally, the present study appears to provide insights for a more comprehensive understanding of Self-Determination Theory. This theory suggests that social environments that do not support individuals’ basic needs frustrate internal motivational sources, negatively affecting individual well-being [83]. The results indicate that excessive social interaction caused by increased working hours can frustrate human functioning when social environments do not provide the necessary conditions to satisfy nurses’ psychological needs, thus decreasing job satisfaction.

## 5. Conclusions

This study aimed to analyze the direct and indirect relationship between workaholism and job stress, with job satisfaction and psychological capital mediating this relationship. The results showed that workaholism contributes to increased job stress, which may affect the positive outcomes in the organizational context. This finding is relevant as there is no consensus in the literature on the nature of the relationship between workaholism and job stress among nurses [19]. Furthermore, the study also found evidence of the mediating role of job satisfaction and psychological capital in the relationship between workaholism and job stress. According to Mensah [68], knowing more about the mechanisms that minimize job stress positively impacts employees’ mental and physical well-being, thereby contributing to improved organizational performance. Therefore, the present study emphasizes that managers and other leaders interested in minimizing job stress could make efforts to improve job satisfaction and the development of psychological capabilities.

## Figures and Tables

**Table 1:** Means, standard deviations and correlations between study variables.

Study Variables	M	SD	1	2	3	4
1. Workaholism	2.75	0.94	(0.74)			
2. Job Satisfaction	3.52	0.91	−0.229 **	(0.81)		
3. Psychological Capital	3.92	0.67	−0.142 **	0.195 **	(0.76)	
4. Job Stress	2.47	1.06	0.310 **	−0.774 **	−0.211 **	(0.88)

*n* = 336. Cronbach’s αs (in parentheses). **. The correlation is significant at the 0.01 level (2-tailed).

**Table 2:** Results of confirmatory factor analysis.

Study Variables	χ^2^	Df	*p*	TLI	CFI	GFI	SRMR	RMSEA
1. Workaholism	24.352	5	<0.001	0.900	0.949	0.971	0.048	0.071
2. Job Satisfaction	88.589	5	<0.001	0.947	0.907	0.914	0.049	0.032
3. Psychological Capital	9.794	5	<0.001	0.979	0.990	0.989	0.068	0.054

## Data Availability

All data will be made available by the author, without undue reservation.

## Public Involvement Statement

Nurses participated voluntarily in this study and received no compensation.

## Guidelines and Standards Statement

This manuscript was drafted against the STROBE checklist appropriate for cross-sectional research.
